# Surgical trial in traumatic intracerebral hemorrhage (STITCH(Trauma)): study protocol for a randomized controlled trial

**DOI:** 10.1186/1745-6215-13-193

**Published:** 2012-10-16

**Authors:** Barbara A Gregson, Elise N Rowan, Patrick M Mitchell, Andy Unterberg, Elaine M McColl, Iain R Chambers, Paul McNamee, A David Mendelow

**Affiliations:** 1 Neurosurgical Trials Unit, 3–4 Claremont Terrace, Newcastle University, Newcastle upon Tyne, NE2 4AE, UK; 2 Department of Neurosurgery, University of Heidelberg, Im Neuenheimer Feld 400, Heidelberg, D-69120, Germany; 3 Institute of Health and Society, Newcastle CTU, 4th Floor, William Leech Building, The Medical School, Framlington Place, Newcastle upon Tyne, NE2 4HH, UK; 4 James Cook University Hospital, Marton Road, Middlesbrough, TS4 3BW, UK; 5 Health Economics Research Unit, Institute of Applied Health Sciences, University of Aberdeen, Polwarth Building, Forester Hill Aberdeen, Scotland, AB25 2ZD, UK

**Keywords:** Head injury, Traumatic intracerebral hemorrhage, Hematoma, Early surgery

## Abstract

**Background:**

Intracranial hemorrhage occurs in over 60% of severe head injuries in one of three types: extradural (EDH); subdural (SDH); and intraparenchymal (TICH). Prompt surgical removal of significant SDH and EDH is established and widely accepted. However, TICH is more common and is found in more than 40% of severe head injuries. It is associated with a worse outcome but the role for surgical removal remains undefined. Surgical practice in the treatment of TICHs differs widely around the world. The aim of early surgery in TICH removal is to prevent secondary brain injury. There have been trials of surgery for spontaneous ICH (including the STICH II trial), but none so far of surgery for TICH.

**Methods/Design:**

The UK National Institutes of Health Research has funded STITCH(Trauma) to determine whether a policy of early surgery in patients with TICH improves outcome compared to a policy of initial conservative treatment. It will include a health economics component and carry out a subgroup analysis of patients undergoing invasive monitoring. This is an international multicenter pragmatic randomized controlled trial.

Patients are eligible if: they are within 48 h of injury; they have evidence of TICH on CT scan with a confluent volume of attenuation significantly raised above that of the background white and grey matter that has a total volume >10 mL; and their treating neurosurgeon is in equipoise.

Patients will be ineligible if they have: a significant surface hematoma (EDH or SDH) requiring surgery; a hemorrhage/contusion located in the cerebellum; three or more separate hematomas fulfilling inclusion criteria; or severe pre-existing physical or mental disability or severe co-morbidity which would lead to poor outcome even if the patient made a full recovery from the head injury.

Patients will be randomized via an independent service. Patients randomized to surgery receive surgery within 12 h. Both groups will be monitored according to standard neurosurgical practice. All patients have a CT scan at 5 days (+/−2 days) to assess changes in hematoma size. Follow-up is by postal questionnaire at 6 and 12 months. The recruitment target is 840 patients.

**Trial registration:**

Current Controlled Trials ISRCTN19321911

## Background

More than 150,000 patients with head injury are admitted to hospital each year in the UK. Of these about 20,000 are serious. One year after a serious head injury 35% of patients are dead or severely disabled. Intracranial hemorrhage occurs in more than 60% of serious head injuries in one or more of three types: extradural; subdural; and intraparenchymal. Prompt surgical removal of significant subdural and extradural hemorrhage is of established and widely accepted value. Intraparenchymal hemorrhage is commoner than both the other types put together and is found in >40% of severe head injuries. It is clearly associated with a worse outcome but the role for surgical removal remains undefined. Several terms are used to describe the condition including traumatic intraparenchymal hemorrhage, traumatic intracerebral hemorrhage (TICH), and contusion. Our own prospectively collected data in over 7,000 head-injured patients in Newcastle has shown that contusions are more common in older head-injured patients and can occur in patients with less severe head injury.

Surgical practice in the treatment of TICHs differs widely. Several issues inform the debate: (a) contused brain does not recover but appears as encephalomalacic brain tissue loss on convalescent phase imaging. This argues that removing TICHs does not increase tissue loss; (b) Extravasated blood is believed to be neurotoxic leading to secondary injury that may be avoided by surgical removal; (c) Larger TICHs may be associated with an ischemic penumbra of brain tissue that could be salvaged; and (d) Some TICHs expand to the point where they cause mass effect resulting in secondary brain injury.

The aim of early surgical TICH removal is to prevent secondary brain injury from these mechanisms. Use of the operation varies around the world. It is more frequently done in Asia than in Europe or North America.

There have been trials of surgery for spontaneous ICH (including the ongoing MRC-funded STICH II study ( http://research.ncl.ac.uk/stich/)) but none so far of surgery for TICH. The Cochrane Review (2nd Edition) has shown benefit from surgical evacuation for spontaneous supratentorial ICH (SICH) [1]. There are differences in the pathogenesis, clinical behavior, and outcome for the two conditions [2]. Patients suffering a TICH tend to be younger by about 15 years on average than patients suffering a spontaneous ICH and therefore the level of disability may have a large effect on their ability to return to work and their economic output. Traumatic ICHs are more likely to be lobar, to be superficial, and to have a medium-sized volume (25 to 65 cc). These differences between the conditions mean that we cannot derive the role of surgery for TICH from results of the 13 published trials of surgery for spontaneous ICH but the STICH trial showed a trend towards better outcome with surgery for the group of spontaneous supratentorial ICH that are most like TICH: superficial hematomas with no intraventricular bleed [3].

We already know that surgery is effective in patients with traumatic EDH and SDH and that early surgery is better than delayed. This is not known for TICH. If early surgery is of benefit to these patients, then implementation of early referral and diagnosis with immediate treatment may reduce death and disability in this specific group of head-injured patients.

Several authors [4-6] have compared surgery with conservative treatment in single center retrospective series and recommended surgery for larger TICHs even if patients were in an apparently good clinical state initially. Matheisen *et al.*[4] found that patients with an admission Glasgow Coma Score of at least 6 and a lesion volume of at least 20 mL who had surgery without previous neurological deterioration had significantly better outcomes than those who did not have surgery or had surgery after deterioration. None of the patients who had surgery before any deterioration died or were vegetative as opposed to 39% of those who had surgery after deterioration and 50% of those who did not have surgery. Choksey *et al.*[5] found that 38% of patients with a low GCS and a volume of the TICH >16 mL who had surgery had a poor outcome compared to 56% of those who did not have surgery. Zumkeller *et al.*[6] found that the poor outcome rate in the operated patients was 29% compared to 59% in the non-operated group. Such associations do not represent true causes and effects or treatment benefits or harm.

Boto *et al.*[7] evaluated the characteristics of severely head-injured patients with basal ganglia TICH and found that they tended to enlarge in the acute post-traumatic period. They found that patients with a TICH of >25 mL and those in whom TICH enlargement or raised intracranial pressure had occurred had the worst outcomes. They suggested that these patients might benefit from more aggressive surgical treatment.

D’Avella *et al.*[8] published a series and suggested that non-comatose patients with smaller TICHs may be treated conservatively but that surgery is indicated for patients with larger TICHs. Most of their comatose patients who were severely injured had a poor outcome whatever treatment was used.

None of these studies involved randomization into surgical and non-surgical groups. They also differed in the characteristics of the parenchymal blood. Such uncontrolled observational studies are potentially misleading and a randomized controlled trial is needed otherwise the wrong conclusion could be drawn.

Guidelines for the Surgical Management of Traumatic Brain Injury were published in 2006 in *Neurosurgery* (2006, **58:**S2-1-62). These confirm that studies in this area have been observational and there is a lack of Class 1 evidence from well-designed randomized controlled trials [9]. Those studies that attempt to compare outcome between surgical and non-surgical groups cannot adequately control for known prognostic variables.

NICE have recommended in the Head Injury Update Full Guideline (2007) that research is needed to develop a consensus on criteria for lesions not currently considered to be surgically significant: namely TICH. This trial (STITCH(TRAUMA)) has been recommended by the latest NICE Head Injury Guideline Development Group.

This study (STITCH(TRAUMA)) is to evaluate the role of early surgical removal of traumatic intracerebral hematomas.

### Objectives

To determine whether a policy of early surgery in patients with traumatic intracerebral hemorrhage improves outcome compared to a policy of initial conservative treatment.

To assess the relative costs and consequences of early surgery *versus* conservative management in UK patients and those in a subgroup of countries covering the likely highest recruiting centers.

To confirm appropriate thresholds for intracranial pressure (ICP) and cerebral perfusion pressure (CPP) for clinical management of head-injured patients with TICH in the subgroup of patients with such monitoring.

## Methods/Design

### Trial design

The Surgical Trial in Traumatic Intracerebral Haemorrhage (STITCH(TRAUMA)) is an international multicenter pragmatic randomized parallel group trial comparing early surgical evacuation of TICH with initial conservative treatment. Only patients for whom the treating neurosurgeon is in equipoise about the benefits of early surgical evacuation compared to initial conservative treatment are eligible for the trial. An independent 24-h telephone and web randomization service based in the Aberdeen Clinical Trials Unit is used. This is backed up by 24-h availability of Trial Investigators who can advise on patient eligibility. Random allocation ensures that the two groups are balanced within a geographic region with a minimization algorithm based on age and severity. Outcome is measured at 6 and 12 months via a postal questionnaire using the extended Glasgow Outcome Scale.

Additional data is collected in those centers that practice invasive brain monitoring of intracranial pressure (ICP) and cerebral perfusion pressure (CPP) to see if there is evidence that such monitoring techniques add value to clinical decision-making. This will give an unbiased assessment of the effect of clot removal or not on ICP/CPP. This analysis will help to evaluate whether monitoring ICP/CPP provides additional information that informs better clinical management (the third objective). Such monitoring is not mandatory for a patient to be enrolled in the trial.

Relevant healthcare costs will be assessed in the UK including length of hospital stay and the costs associated with surgical treatment (theater time, consumables, overheads); healthcare resource use outside of hospital (for example, district nurse, physiotherapy) together with productivity costs arising from absence from work; and additional costs for family members through extra caring responsibilities. Consequences will be measured by combining data on quality of life, measured using the EQ-5D with survival to generate Quality Adjusted Life Years (QALYs).

### Screening logs

Screening logs are maintained by each participating center to record: the patients admitted to the neurosurgical unit with any traumatic ICH; whether they are eligible for the trial or not and whether they are recruited or not (and if not, why not, if the reason can be ascertained). These will be used to provide a context for the study, to monitor recruitment rates, and as the basis for constructing the CONSORT diagram for reporting the trial.

### Center eligibility

The centers recruited are those already collaborating successfully with the team in other studies (STICH, STICH II, RescueICP) plus those identified by the various networks: TARN (Trauma Audit and Research Network), EBIC (European Brain Injury Consortium) and EMN (Euroacademia Multidisciplinaria Neurotraumatologica), BrainIT, EANS (European Association of Neurosurgical Societies), GNAMED (Scottish and Newcastle Neurosurgery Research Group), SBNS (Society of British Neurological Surgeons), and BNRG (British Neurosurgery Research Group).

Only centers that can demonstrate effective trial experience and previous adherence to trial guidelines with high follow-up rates are eligible to take part. In order to be eligible a center must be able to recruit a minimum of one patient per year. They must be able to communicate with the research team. (At least one member of the local team must be proficient in English and provide contact details where they can be reached easily to support the local center and respond to the trial management team in Newcastle.) They must be able to provide CT scans of sufficient quality to the study centre in Newcastle. They must be able to arrange follow-up for patients with limited literacy.

Each center is required to obtain ethical approval and other permissions as needed to conform with local and national legislation and research governance frameworks and to provide documentary evidence to the trial management team that these permissions are in place, prior to site registration and initiation. Each site is also required to sign an agreement with the sponsor (Newcastle upon Tyne Hospitals NHS Foundation Trust) and the contractor (Newcastle University). Applications by the lead collaborator in each center for ethical approval (or SSA in the UK) are supported by the trial manager and the clinical lead for the center and country in which the center is located. Also within the UK, R&D approval is sought in respect of all participating centers and the study is open to audit (‘for cause’ or as part of the routine 10% check) by the appropriate research governance teams in the participating Trusts. A member of the study team visits centers with high volume recruitment or where there are concerns about patient eligibility (identified by central monitoring) to confirm patient existence and monitor adherence to the trial protocol, against pre-determined, risk-based criteria (we do not anticipate conducting 100% site data verification).

### Approval to start

MREC approval for the study was obtained from Southampton Multicentre Research Ethics Committee. Appropriate local ethical approval is sought from each participating center in the study with proof of the approval forwarded to the trial coordinating office before recruitment can be started. The trial is conducted according to local ethical and Research and Development procedures. An agreement is signed between the sponsor (Newcastle upon Tyne NHS Hospitals Foundation Trust), the holder of the study funding (Newcastle University) and the hospital center prior to commencing the study at the center.

### Patient recruitment

All appropriate patients who are considered for STITCH(TRAUMA) must have a CT scan to confirm the diagnosis and the size and location of the hematoma. Any clotting or coagulation problems must be corrected prior to randomization in line with standard clinical practice.

#### Inclusion criteria

• Adults aged 14 years or over

• Evidence of a TICH on CT with a confluent volume of attenuation significantly raised above that of the background white and grey matter that has a total volume >10 mL calculated by (width × height × length)/2 in cm

• Within 48 h of head injury

• Clinical equipoise: only patients for whom the responsible neurosurgeon is uncertain about the benefits of either treatment are eligible

#### Exclusion criteria

• A significant surface hematoma (EDH or SDH) requiring surgery (the indications for intervention for these patients are already very well defined)

• Three or more separate hematomas fulfilling inclusion criteria

• If the hemorrhage/contusion is located in the cerebellum

• If surgery cannot be performed within 12 h of randomization

• Severe pre-existing physical or mental disability or severe co-morbidity which would lead to a poor outcome even if the patient made a full recovery from the head injury (examples would be a high level of dependence before the injury or severe irreversible associated injury such as complete spinal cord injury)

• Permanent residence outside a study country preventing follow-up

• Patient and/or relative has a strong preference for one treatment modality

There is no specified upper age limit. The need for clinical equipoise and explicit exclusion of patients with severe pre-existing physical or mental disability or severe co-morbidity which might lead to a poor outcome even if the patient made a good recovery from the head injury excludes the older less able patient while allowing a fit older person to be included. Hematoma rates are known to be more common in the older head-injured patient.

#### Consent procedure

Written witnessed informed consent of the patient or relative must be obtained by trained neurosurgical staff prior to randomization. The member of neurosurgical staff provides a written information sheet and allows as much time as possible to discuss the options. One copy of the consent form is given to the patient, one is filed in the patient notes, and one is filed with the trial documentation. If the patient is unable to give consent themselves due to the nature of the hemorrhage a personal representative is approached to give consent on behalf of the patient. The personal representative is the person with a close personal relationship with the patient who is themselves capable and willing to consent on behalf of the patient. (If the patient is unable to consent and the closest relative is not available the patient cannot be included in the study.)*

* In Scotland, if proxy consent is necessary this should be obtained from the welfare guardian or, if there is none, from the nearest relative.

### Randomization (treatment allocation)

Before randomization, a one-page form is completed by the responsible neurosurgeon recording demographic (age, gender) and TICH characteristics (site, side, ABC measures to define volume) and status at randomization (pupils equal and reacting or not). The clinician either telephones the independent 24-h telephone randomization service or accesses the randomization website and enters the randomization information. At the end of the randomization phone call/web data entry process the neurosurgeon is informed of the patient identifier number for the trial and the treatment group the patient is allocated to. The neurosurgeon records this information on the randomization form and then faxes the form to the STITCH(TRAUMA) Office. If the site has problems contacting the randomization service they are able to contact a member of the project team using the study backup number.

The data manager checks this information against the information received from the randomization center and enters the data into an anonymized password protected database. A list of patient names and study numbers are kept in a separate file to ensure patient confidentiality is maintained.

Allocation is stratified by geographic region, with a minimization algorithm based on age group and severity (as measured by whether the pupils are equal and reacting or not) and with a random component (that is, with probability of 80%).

### Trial interventions

The two trial interventions are: (1) early evacuation of the hematoma by a method of the surgeon’s choice (within 12 h of randomization), combined with appropriate best medical treatment; or (2) best medical treatment combined with delayed (>12 h after randomization) evacuation if it becomes appropriate later. Both groups are monitored according to standard neurosurgical practice.

If the patient is randomized to early surgery this should be undertaken as soon as possible and within 12 h of randomization.

Best medical treatment may include (depending on the practices within the center) monitoring of ICP or other modalities and management of metabolism, sodium osmotic pressure, temperature, and blood gasses.

All patients also have an additional CT scan at about 5 days (+/−2 days) to assess changes in the hematoma size with and without surgery. This will enable us to demonstrate the proportion of the clot removed by surgery or the changes in volume of the clot without surgery.

### Compliance

Patients or their relatives may withdraw consent for an operation, or conversely request an operation after randomization, thereby leading to crossover between the arms. These are rare events but in surgical trials it is common for the patient’s condition to change over time and a patient randomized to initial conservative treatment may deteriorate and require surgery later. Such crossovers and the reasons for them are documented. Information is collected about the status (GCS and focal signs) of patients through the first 5 days of their trial progress and ICP/CPP measures in invasively monitored patients in order to be able to describe the change in status that leads to a change in equipoise for the treating neurosurgeon, and subsequent surgery in patients initially randomized to conservative treatment.

Compliance with treatment allocation is monitored by the data manager.

In surgical trials patients allocated to the non-surgical arm of the trial may later deteriorate and surgeons may intervene. This was the case in the MRC-funded STICH trial [3], in trials of cardiac surgery compared with angioplasty, in the MRC-funded back pain trial [10] and in the SPORT trials [11]. These crossover rates to surgery were 26%, 28%, 28%, and 30%, respectively. While surgical trials will always have such crossovers when surgeons perceive that there is value in operating on patients who deteriorate after initial randomization into the conservative limb of the trial, we must understand, monitor, and report the rates of such crossovers.

The aim is to achieve as high compliance as possible but experience and the above literature suggest that it is neither practical nor ethical to have 100% compliance with conservative treatment. During the recruitment of centers and at investigator meetings the importance of clinical equipoise and minimizing crossovers is emphasized and any crossover occurring within 12 h of randomization is investigated. Centers exhibiting high crossover rates may be withdrawn from the study.

### Data collection

To preserve confidentiality all patients are allocated a unique study identifier during the randomization process which is used on all data collection forms and questionnaires. Only a limited number of members of the research team are able to link this identifier to patient identifiable details. This is necessary in order to carry out centralized follow-up.

All study documentation is held in secure offices and the study research team operate to a signed code of confidentiality. All data are entered into an anonymized password-protected database by the data manager. Paper copies of questionnaires are kept in locked cabinets in a locked room.

Any previously collected data are retained for patients who subsequently withdraw from the trial. This data is anonymized and kept confidential.

Trial documentation will be kept for 15 years after publication of the final paper/report from this study.

#### Randomization form

This provides baseline information and is required in order to randomize the patient.

#### Two-week/discharge form

At 2 weeks after randomization or at discharge or death (whichever occurs first) the discharge/2-week form is completed by the responsible neurosurgeon or research nurse. This form records the date, the event that triggers the form, and the patient’s status at that time, whether the patient has had surgery (and why if randomized to initial conservative treatment or why not if randomized to early surgery), the patient’s GCS and localizing features for the 5 days following randomization, the occurrence of any adverse events (including death, pulmonary embolism, deep vein thrombosis, surgical site infection) following randomization, past medical history, and status prior to the ictus. This form together with copies of the randomization CT scan and the 5-day post-randomization CT scan (as detailed below) should be sent to the STITCH(Trauma) office at the Neurosurgical Trials Unit in Newcastle UK within 2 weeks. The data manager enters the data into the anonymized password-protected database.

#### CT scans

Copies of two CT scans are required: the diagnostic CT scan prior to randomization and a 5-day scan. All patients have undergone a diagnostic CT scan as standard practice. The 5-day scan is performed between 3 and 7 days after randomization. Many patients receive this as part of standard treatment and the study accepts and uses any scan taken for clinical purposes during this period. Only patients who do not receive such a scan during this period require an additional scan.

The preferred scan is a CT scan with volume acquisition 32 × 0.5 mm (or equivalent); 120 Kv 400 mA (or equivalent); 220 FOV. The angle should be parallel with the anterior cranial fossa, coverage from base of skull to vertex; reconstruct 5 mm whole head, soft tissue filter.

The preferred method of sending CT scans is in DICOM compatible format. DICOM images (on separate CDs for the two time points) are sent anonymized with patient identifier. They are checked by the data manager initially on receipt at the STITCH(Trauma) office to ensure that the hematoma characteristics at randomization conform to the required inclusion criteria. Where protocol deviations are suspected the data manager arranges for the scan to be viewed by a trained reader immediately and if their suspicions are confirmed the center is contacted immediately to prevent repetitions.

The data manager loads the scans into a specialized password-protected scan management program. The scans are then allocated a separate randomly created identifier by the data manager, so that it is not possible for the reader to identify the before and after scans of the same patient. The scans are stored in locked cabinets. A separate list identifying patient identifier and scan identifier is kept by the data manager.

The CT scans are analyzed subsequently by trained readers using the scan management program. Their passwords only give access to scans blinded to treatment group and patient identity following a defined protocol.

#### Follow-up

Postal follow-up occurs at 6 and 12 months using questionnaires translated into the appropriate language. The patient’s GP (in the UK) or consultant (outside the UK) is contacted at 4.5 months to check that the patient is alive and to confirm his/her place of residence. At this time the GP/Consultant is also requested to complete a major adverse events form. The 6-month outcome questionnaire is mailed to the patient at 5 months for completion by the patient or relative if the patient is unable to complete it themselves. If necessary a reminder is sent at 6 months and telephone follow-up at 7 months by ‘blinded’ clerical or nursing staff to enhance response rates. In countries where the postal system is poor, patients are requested to attend a follow-up clinic at which the questionnaire is distributed and collected. In countries where literacy or language/dialect are problematic a ‘blinded’ interviewer administers the questionnaire. This methodology has been used to good effect in previous studies: STICH and STICH II.

**Figure 1:**
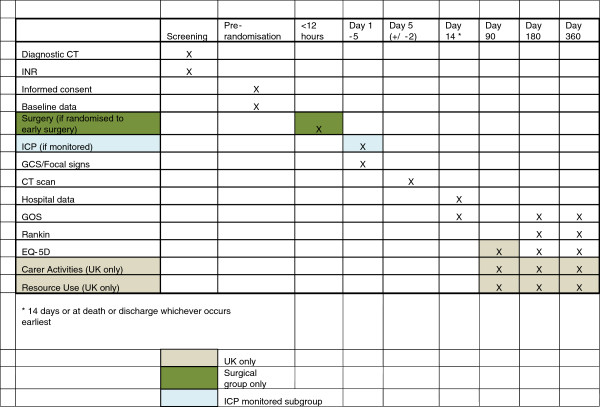
Chart showing study timetable for treatment of patients.

The costs associated with surgical treatment (theater time, consumables, overheads) will be collected from published resources and local cost surveys undertaken by the study health economist. Length of stay, healthcare resource use outside of hospital, together with productivity costs arising from absence from work, and additional costs for family members through extra caring responsibilities are collected using the additional 3-month postal questionnaire and extended 6-month and 12-month postal questionnaires in the UK. Consequences will be measured by combining data on quality of life with survival to generate Quality Adjusted Life Years. This will include measurement of healthcare costs, quality of life (EQ-5D), work absence (WHO Health and Performance Questionnaire-Clinical Trial Version), and carer activities (measured by Discrete Choice Experiment developed by HERU). EQ-5D and survival are collected for all patients by the postal outcome questionnaires in order to generate QALYS for the whole study and for a UK-only analysis (Figure 1).

### Serious adverse events

Serious adverse events (SAEs) are recorded on the Major Adverse Events form. Serious adverse events are adverse experiences that result in any of the following outcomes: death; life-threatening events; requirement for inpatient hospitalization or prolongation of existing hospitalization; or persistent or significant disability or incapacity.

All SAEs should be reported to the STITCH Office within 7 days of the local investigator becoming aware of the event and to the local ethics committee or other regulatory bodies as required.

### Outcome measures

Primary: Unfavorable outcome will be death or severe disability which will be defined using a prognosis based eight-point Glasgow Outcome Scale/ Modified Rankin Scale [3,12].

Secondary: Rankin, EQ-5D, Mortality, Survival, Major Adverse Events (death, pulmonary embolism or deep vein thrombosis, infection, re-hemorrhage), QALYs, total healthcare costs, social costs.

The Glasgow Outcome Scale is the specific measure for head injury and the eight-point scale provides more sensitivity than the five-point scale. For patients with a very poor prognosis an outcome of good recovery, moderate disability or upper severe disability would be regarded as a favorable outcome. For patients with a better prognosis favorable outcome would be good recovery or moderate disability. A structured postal version has been developed [13]. The Rankin scale is widely used as a functional outcome measure in stroke and allows comparison of results between this study of patients with traumatic ICH and studies of patients with spontaneous ICH. EQ-5D is the standard measure of quality of life incorporating a utility value and has been developed in many languages.

### Sample size

Previous studies have suggested a favorable outcome in the non-operated group of about 40% and a favorable outcome in the surgical group of about 60% to 70%. However this was in observational studies. Assuming a favorable outcome (good recovery or moderate disability on the Glasgow Outcome Scale) of 50% from conservative treatment a total sample size of 776 would be required to show a 10% benefit (that is, 50% *vs.* 60%) from surgery (2p <0.05) with 80% power. A safety margin of 9.5% is built in to allow for loss to follow-up making a total sample size of 840 to be recruited and randomized (420 per arm).

In order to achieve this sample size in a reasonable time span and to provide robust evidence it is necessary to recruit patients from outside UK. In England and Wales there are only 30 neurosurgical units and only one-third of these participate in randomized controlled trials. Experience with interested neurosurgical centers in previous studies has shown that about 25% of recruited centers fail to recruit any patients and a further 25% only recruit one or two patients. The best recruiting centers will recruit about 10 patients per year so to complete patient recruitment within the timescale we will approach at least 150 centers.

Loss to follow-up is kept as low as possible. In the STICH study the loss was about 5%. In STITCH(TRAUMA) the population is a little younger and likely to be more mobile; however, we carry out more checks and implement procedures that we have developed to minimize loss to follow-up. Methods of follow-up are adapted to those most likely to be successful within each country and center according to local population and care characteristics. Centers that achieve poor follow-up are monitored closely and may be withdrawn from the study if they are unable to locate patients for 6-month follow-up. We require residence in any study country as an eligibility criterion so patients who suffer a head injury whilst on holiday and might be lost to follow-up are not eligible and are not included.

### Blinding

It is not possible to blind either patients or treating surgeons as to when the patient has had surgery or whether they have had surgery. To minimize possible sources of bias, randomization is undertaken centrally, thus ensuring concealment of allocation from the enrolling clinician, patient, and relatives. All patients randomized, for whom outcome data can be collected, will be included in the analysis by intention to treat. The multidisciplinary team in the co-ordinating center and the principal investigators will be blinded to the results until after the data set is locked following receipt of the final outcome questionnaire. Only the data manager will have access to ‘unblinded’ data.

### Statistical analysis

Analysis will be on an ‘intention-to-treat’ basis. The primary analysis will be a simple categorical frequency comparison using the uncorrected chi-squared test for prognosis-based [13,14] favorable and unfavorable outcomes at 6 months. Patients with a good prognosis will be categorized as having a favorable outcome if they achieve good recovery or moderate disability on the Glasgow Outcome Scale. Patients with a poor prognosis will be categorized as having a favorable outcome if they achieve good recovery, moderate disability, or upper severe disability on the extended Glasgow Outcome Scale. Logistic regression analysis will be undertaken to adjust for covariates. Secondary outcomes will also be analyzed using the prognosis based method as specified in STICH [3].

Given the likelihood of a proportion of crossovers, a secondary sensitivity analysis of per-treatment as well as an analysis considering crossovers to surgery as failed medical treatment will be undertaken. Further analyses of factors that drive crossovers as well as per-protocol and per-treatment analyses will be conducted to investigate the effect of crossovers.

Any subgroup analyses will be based on tests of interaction. The predefined subgroups (all of which will be considered exploratory, since the study is not powered for formal subgroup analyses) include the following: age; hematoma volume; Glasgow Coma Score; time from injury to randomization; severity of neurological deficit; pupils equal and reacting or not; planned method of hematoma removal; patients with invasive monitoring (ICP/CPP); anticoagulation status.

Interim analyses are conducted at intervals predetermined by the DMEC. The results of interim analyses are strictly confidential and the trial will only be stopped early if one or other treatment policy shows an advantage at a very high significance level, or if recruitment rates are unexpectedly low.

Deterministic and probabilistic sensitivity analyses will be conducted to consider the importance of individual parameters and assumptions in determining cost-effectiveness. This will include the effect of time horizon, variation in unit costs across centers, and quality of life values. Bootstrapped-generated differences in costs and effectiveness between strategies will be computed, and results presented using cost-effectiveness acceptability curves (CEACs).

Receiver-operating curves will be used to investigate appropriate thresholds of ICP and CPP for treatment as they have been used previously in pediatric studies [15].

### Ethical issues and research governance

#### Risks and anticipated benefits for trial participants and society

Risks and benefits for trial participants - the risks from undergoing surgery include risks of complications due to undergoing a general anesthetic and surgery; however, the risks of undergoing early surgery may be equivalent to the risks of delaying surgery. Only those patients for whom the treating clinician, patient, and relative are in equipoise regarding early surgery *vs.* conservative management are enrolled in the trial.

Anticipated benefit for society is that of improved outcome for patients in the future. The results will inform decision-making, permitting evidence-based policies to be developed for the management of traumatic ICH. If surgery is shown to be ineffective, then cost savings can be made by avoiding surgery. If surgery is shown to be effective, then better outcomes will be achieved for the patient together with reduced rehabilitation and recovery costs to the NHS and the patients and their families.

### Trial committees

#### Data monitoring and ethics committee

In order to monitor accumulating data on patient safety and treatment benefit an independent data monitoring and ethics committee (DMEC) was established. The DMEC considers data from interim analyses and reports to the Trial Steering Committee. A written charter was developed and agreed prior to the first DMEC meeting. Interim analyses are strictly confidential and the committee will only recommend stopping the trial early if one or other treatment shows an advantage at a very high significance level.

#### Management committee

This group meets weekly to monitor progress and compliance.

#### Trial steering committee

Independent oversight of the study is provided by a Trial Steering Committee (TSC) which meets every 6 months. The Trial Steering Committee provides overall supervision of the trial on behalf of the HTA. It considers progress of the trial (in particular, success in site and patient recruitment), adherence to the protocol, patient safety, and consideration of new information. The trial is conducted according to the standards set out in the MRC Guidelines for Good Clinical Practice. A written charter was developed and agreed prior to the first TSC meeting.

### Roles and responsibilities

#### Principal investigators and trial team

Professor A D Mendelow has overall responsibility for the trial. He is also responsible for disseminating information about the trial, recruiting centers, and for writing and publication of the results.

Dr B A Gregson is responsible for the overall statistical validity of the trial and day-to-day conduct of the trial including availability of coordinating advice in Newcastle. She is also responsible for preparation of protocols and questionnaires, for MREC application, for preparing annual reports to HTA and Ethics committees, for communication and dissemination of information to centers, for monitoring centers, for data analysis, and for writing up of results.

Mr P Mitchell is responsible for recruiting centers and for analysis and publication of results.

Professor Elaine McColl is responsible for ensuring that the trial is run according to GCP guidelines and advises on overall trial conduct and project management.

Dr Iain Chambers is responsible for ensuring the quality of ICP and CPP data collected and for the analysis of this data.

Dr Paul McNamee is responsible for the economic validity of the trial; he is responsible for the design of the economic component of the trial and over sees the economic data collection and analysis.

The trial manager is responsible for ensuring ethics approvals and agreements are in place in all centers, negotiating as required between contracts personnel, to maintain a website to encourage site and patient recruitment, to provide reports to trial management and steering committees, to the funder and to the research ethics committees as required, to monitor compliance and to communicate with the centers.

The data manager is responsible for maintaining computerized databases containing all data related to the trial, for the quality of computerized information, for conducting preliminary analyses and preparing reports for the DMEC, for providing information to the applicants, and for preparing monthly newsletters.

The trial secretary is responsible for all trial correspondence in relation to the trial, for sending postal questionnaires and reminders, for the organization of investigator meetings and travel for monitoring, maintaining telephone and fax communications, preparing quarterly newsletters and publications, and reimbursing centers.

The health economist is responsible for undertaking the collection and analysis of economic data.

#### Responsibilities of national investigators

In countries with multiple centers one center investigator fulfills the role of National Investigator. National investigators are responsible for obtaining national ethical approval and other permissions as required, for ensuring that documentation is translated from English as required, for identifying suitable centers within their country, for encouraging recruitment, and acting as a liaison person between the STITCH(TRAUMA) team and the center if required.

#### Responsibilities of center investigators

Each center agrees to follow the protocol. They provide and update when necessary full address and contact details. Within each center there is at least one named collaborator who is responsible for the conduct of the trial in his/her center and in particular for: local ethical applications and applications for other permissions as required; disseminating information about the trial within the center; maintaining local trial documentation, including site files, delegation logs, et cetera; identifying suitable patients; ensuring all case report forms are completed and returned to the STITCH office in Newcastle expeditiously; ensuring copies of CT scans are provided to STITCH office in Newcastle expeditiously; ensuring follow-up is obtained in the center; attending investigator meetings (in person or via video- or teleconference); facilitating center monitoring; commenting on the final report.

Centers receive a monitoring visit as required either after recruiting at least 10 patients or if there is a perceived need ( Additional file 1).

### Trial coordinating centre details

Address: STITCH(Trauma), Neurosurgical Trials Unit, 3–4 Claremont Terrace, Newcastle University, Newcastle upon Tyne, NE2 4AE, UK.

Email: trauma.STITCH@ncl.ac.uk

Phone: +44 191 222 5764

Fax: +44 191 222 5762

#### Trial website

http://research.ncl.ac.uk/trauma.stitch

#### Trial randomization service (telephone and web service)

Aberdeen HSRU: +44 (0) 1224 273661

https://viis.abdn.ac.uk/HSRU/stitch

#### Principal Investigators

A David Mendelow, MB BCh PhD FRCS

Barbara A Gregson, BSc PhD FSS

Patrick M Mitchell, BA MB BChir BSc FRCS PhD

Andy Unterberg, MD, PhD

Elaine M McColl, BA MSc PhD

Iain R Chambers, BSc PhD CEng FIPEM

Paul McNamee, MA MSc PhD

#### Trial Steering Committee

Mr J Steers (Independent Chairman)

Dr Andy Vail (Statistician)

Dr D Birchall (Neuroradiologist - Independent Member)

Mr Jake Timothy (Neurosurgeon - Independent Member)

Professor Luke Vale (Health Economist - Independent Member)

Mr A White - Headway

Mr D O’Meara - UKABIF

Professor AD Mendelow

Dr BA Gregson

Mr PM Mitchell

Dr A Unterberg

Professor EM McColl

Dr IR Chambers

Dr P McNamee

#### Data Monitoring Committee

Mr P Hutchinson (Chairman)

Professor GD Murray

Dr A Gholkar

#### Trial Management Team

Dr Barbara A Gregson (Trial Director)

Professor A David Mendelow (Chief Investigator)

Dr Elise Rowan (Trial Manager)

Dr Richard Francis (Data Manager)

(Economist)

Miss Courtenay Howe (Trial Secretary)

Professor Elaine McColl

Mr Patrick Mitchell

#### Project sponsorship, ethics, and registration details

Sponsor: Newcastle upon Tyne Hospitals NHS Foundation Trust

Sponsor No: 4792

Funding Source: NIHR HTA 07/37/16

HTA Grant No: 07/37/16

ISRCTN No: ISRCTN19321911

Host No: BH080479

IRAS No: 09/H0502/68

## Trial status

At the time of submission of this protocol (June 2012), this study is ongoing.

## Abbreviations

CPP: Cerebral perfusion pressure; CT: Computed tomography scan; CTU: Clinical trials unit; DMEC: Data monitoring and ethics committee; EDH: Extradural hematoma; GCP: Good clinical practice; GCS: Glasgow Coma Score; GOS: Glasgow Outcome Scale; ICP: Intracranial pressure; ICH: Intracerebral hemorrhage; SAE: Serious adverse event; SDH: Subdural hematoma; TICH: Traumatic intracerebral hemorrhage including contusion; TSC: Trial steering committee.

## Competing interests

There are no conflicts of interest.

## Authors' contributions

ADM is the Chief Investigator for STITCH(Trauma) and leads the project. BAG is the trial director and was responsible for the initial development of the protocol along with ADM. BAG advised on the statistical, sample size, and data analysis sections in particular. ENR is the trial manager, coordinated development of trial documentation and UK ethics approval, and took the main responsibility for preparing and editing this article for journal publication. PMM, AU, EMM, IRC, and PMcN are all co-PIs on the project and have made major contributions to developing the protocol prior to the start of the trial. All authors are also active members of the trial management team or trial steering committee and have commented on this article. All authors read and approved the final manuscript.

## Supplementary Material

Additional file 1 STITCH(Trauma) Participating Centers (as of 12 June 2012). Click here for file
