# Supplementary material for: Surgical trial in traumatic intracerebral hemorrhage (STITCH(Trauma)): study protocol for a randomized controlled trial
Source: Trials. 2012 Oct 16;13:193. doi: 10.1186/1745-6215-13-193 (PMC3488505; doi:10.1186/1745-6215-13-193)
Supplement: Additional file 1 — STITCH(Trauma) Participating Centers (as of 12 June 2012). [file 1745-6215-13-193-S1.docx]

**STITCH (Trauma) Participating Centres (as of 12^th^ June 2012)**

**ARMENIA –** Yerevan State Medical University, Dr Ruben Fanarjyan

**BULGARIA –** University Hospital Pirogov, Sofia, Associate Professor Nikolay Gabrovsky

**CANADA –** St Michael’s Hospital, Toronto, Professor Loch MacDonald

**CHINA –** Beijing Tiantan Hospital, Professor Zhao Jizong

Huashan Hospital, Shanghai, Dr Liangfu Zhou

Tianjin Medical University General Hospital, Professor Shuyuan Yang

**CZECH REPUBLIC –** University Hospital Brno, Professor Martin Smrčka

**EGYPT –** Alexandria Main University Hospital, Professor Osama S. Abdelaziz

Zagazig University Hospital, Dr Mohamed Barakat

**GERMANY –** Klinikum Bogenhausen, Munich, Professor Christianto B. Lumenta

University Clinic Jena, Professor Rolf Kalff

University Hospital Heidelberg, Professor Andreas Unterberg

University of Ulm Medical School, Professor Marc-Eric Halatsch

University of Ulm – Günzburg, Professor Marc-Eric Halatsch

**HUNGARY –** University of Szeged, Professor Pal Barzo

**INDIA –** Acharya Vinoba Bhave Rural Hospital, Maharashtra, Dr Anand Kakani

All India Institute of Medical Sciences, New Delhi, Dr Sarat Chandra

AMRI Hospitals – Dhakuria, Calcutta, Professor R N Bhattacharya

BGS Global Hospital, Bangalore, Dr Shailesh Rao

Care Hospital, Visakhapatnam, Dr P V Ramana

Christian Medical College & Hospital, Ludhiana, Dr Sarvpreet Singh Grewal

CSM Medical University, Lucknow, Dr Sunil Singh

Fortis Malar Hospital, Chennai, Dr Venkat Prasanna

Himalayan Institute of Medical Sciences, Dehradun, Professor Charitesh Gupta

Kamineni Life Sciences, Hyderabad, Dr Subodh Raju

Kerala Institute of Medical Sciences, Kerala, Dr Moni K Vinod

MM Institute of Medical Sciences & Research, Haryana, Dr Amit Agrawal

Mysore Clinisearch, Nazarbad, Dr Anil Sangli

NIMHANS, Bangalore, Dr Jagath lal Gangadharan

**ITALY –** Catholic University Gemelli Hospital, Rome, Professor Carmelo Anile

**LATVIA –** Pauls Stradins Clinical University Hospital, Riga, Dr Egils Valeinis

**LITHUNIA –** Kaunas University Hospital, Professor Rimantas Vilcinis

Klaipeda Hospital, Dr Antanas Gvazdaitis

**MALAYSIA –** Hospital of Universiti Sains Malaysia, Prof Dr Jafri Malin Abdullah

Hospital Sultanah Aminah Johor Bahru, Dr Arshad Ali

**NEPAL –** Neuro Hospital, Biratnagar, Dr Yam Roka

**PAKISTAN –** Northwest General Hospital & Research Centre, Peshawar, Dr Tariq Khan

**ROMANIA –** Cluj County Emergency Hospital, Professor Stefan Florian

County Hospital, Timisoara, Dr Horia Ples

Emergency Hospital St. Pantelimon, Bucharest, Associate Professor Danil Adam

**SPAIN –** Hospital Universitario Rio Hortega, Valladolid, Dr Rosario Sarabia

University Hospital Marquès de Valdecilla, Santander, Professor Alfonso Vázquez-Barquero

**UK –** Leeds General Infirmary, Mr Jake Timothy

Royal Victoria Infirmary, Newcastle, Mr Patrick Mitchell

Ninewell’s Hospital, Dundee, Professor Sam Eljamel

Princess Royal Hospital, Haywards Heath, Mr Giles Critchley

Southampton General Hospital, Mr Diederik Bulters

St George’s Hospital, London, Professor B A Bell

The National Hospital for Neurology and Neurosurgery, Mr George Samandouras

**USA –** Albany Medical College, New York, Dr John German

Legacy Emanuel Hospital & Health Center, Portland, Dr Jeff Chen

Pennsylvania State University, Dr Philip Villanueva

Temple University, Philadelphia, Professor Christopher Loftus

University of Louisville, Kentucky, Dr Jonathan Hodes
